# The treatment value of IL-1β monoclonal antibody under the targeting location of alpha-methyl-l-tryptophan and superparamagnetic iron oxide nanoparticles in an acute temporal lobe epilepsy model

**DOI:** 10.1186/s12967-018-1712-3

**Published:** 2018-12-04

**Authors:** Yanli Wang, Yanling Wang, Ran Sun, Xingrao Wu, Xu Chu, Shuhu Zhou, Xibin Hu, Lingyun Gao, Qingxia Kong

**Affiliations:** 1 Department of Neurology, Affiliated Hospital of Jining Medical University, Jining, China; 2 Department of Magnetic Resonance Imaging, Affiliated Hospital of Jining Medical University, Jining, China

**Keywords:** SPIONs, AMT, Anti-IL-1β-mAb, Temporal lobe epilepsy, Targeting location, Targeting therapy

## Abstract

**Background:**

Temporal lobe epilepsy (TLE) is a common and often refractory brain disease that is closely correlated with inflammation. Alpha-methyl-l-tryptophan (AMT) is recognized as a surrogate marker for epilepsy, characterized by high uptake in the epileptic focus. There are many advantages of using the magnetic targeting drug delivery system of superparamagnetic iron oxide nanoparticles (SPIONs) to treat many diseases, including epilepsy. We hypothesized that AMT and an IL-1β monoclonal antibody (anti-IL-1β mAb) chelated to SPIONs would utilize the unique advantages of SPIONs and AMT to deliver the anti-IL-1β mAb across the blood–brain barrier (BBB) as a targeted therapy.

**Methods:**

Acute TLE was induced in 30 rats via treatment with lithium-chloride pilocarpine. The effects of plain-SPIONs, anti-IL-1β-mAb-SPIONs, or AMT-anti-IL-1β-mAb-SPIONs on seizure onset were assessed 48 h later. Perl’s iron staining, Nissl staining, immunofluorescence staining and western blotting were performed after magnetic resonance imaging examination.

**Results:**

The imaging and histopathology in combination with the molecular biology findings showed that AMT-anti-IL-1β-mAb-SPIONs were more likely to penetrate the BBB in the acute TLE model to reach the targeting location and deliver a therapeutic effect than plain-SPIONs and anti-IL-1β-mAb-SPIONs.

**Conclusions:**

This study demonstrated the significance of anti-IL-1β-mAb treatment in acute TLE with respect to the unique advantages of SPIONs and the active location-targeting characteristic of AMT.

## Background

Epilepsy is a common chronic brain disease. Approximately 25% of patients with epilepsy cannot be relieved of their symptoms by conventional drug therapy and will develop refractory epilepsy of which 75% of these cases constitute temporal lobe epilepsy (TLE) [1]. TLE is characterized by recurrent seizures with pathological features such as hippocampal sclerosis and neuronal network alterations [2, 3]. Studies have emphasized that inflammation plays an important role in epilepsy, and seizures are known to promote molecular and structural changes [4]. The inflammatory response can reduce the threshold of epileptic seizures as well as increase the excitability of neurons, damage the blood brain barrier (BBB), and mediate neuronal apoptosis and synaptic remodelling [5]. Interleukin-1β (IL-1β) is the predominant inflammatory factor involved in this process. The level of IL-1β in the brain tissue of patients was positively correlated with the severity of preoperative epilepsy [6], and a clinical study revealed the expression of IL-1β/IL-1R1 in the glial cells and neurons of drug-refractory epilepsy patients [7]. Continuous activation of the IL-1β system causes epilepsy and inflammation, which form a positive feedback loop whereby seizures cause inflammation and inflammation leads to nerve cell excitability and seizures. However, the presence of the BBB prevents the delivery of diagnostic and therapeutic agents to the brain [8]. As a result, fat-insoluble drugs with molecular masses larger than 600 Da are difficult to deliver. Thus, the search for new drugs that can penetrate the BBB has become a research hotspot.

It is desirable that the materials used as drug carriers have both targeted and controlled drug delivery functions, which would not only improve the efficiency of the drugs but also greatly reduce the side effects of the drugs [9]. Drug-loaded magnetic nanoparticles can act accurately on a lesion through an external magnetic field, which can improve the drug concentration in the targeting area and reduce damage to normal tissue, allowing researchers to track the process and distribution of drug delivery in vivo through magnetic resonance imaging (MRI). Superparamagnetic iron oxide nanoparticles (SPIONs) have demonstrated significant advantages as a drug delivery system. Under the action of an alternating magnetic field, SPIONs absorb energy to generate heat energy, which can also control drug release [10]. Through targeting the specific ligand that can be combined with the receptor of the target cell, SPIONs can be directed to the specific cell to play a role in reducing damage to normal cells and actively targeting the lesion. According to the different targeting molecules, the ligand can be divided into antibodies, receptors and others [11–13]. The delivery effects of SPIONs depends on the size of their magnetic particles, and good targeting is observed for magnetic particles of 10–30 nm in size and shoes with a pore structure that is more conducive to drug loading and release.

Neurology experts have found that changing the ligand of nanoparticles can help to target the seizure focus accurately [7]. The present ligand study focused on fluorine-labelled deoxyribose, flumazenil, and alpha-methyl-l-tryptophan (AMT). AMT is a synthetic amino acid and is recognized as a surrogate marker for epilepsy; it was first used in tuberous sclerosis complex (TSC) epilepsy patients [14]. AMT has high uptake in the epileptic focus [15–17] and it can help to distinguish between epileptogenic and non-epileptogenic regions in children and to evaluate surgeries on tuberous sclerosis and especially multifocal cortical dysplasia [18]. Indeed, through the use of positron emission tomography (PET) technology, removing high AMT metabolic zones from children with epilepsy nodular sclerosis has been shown to stop their seizures [19] AMT has been used as a PET ligand to identify epileptogenic tissues in several epilepsy conditions [20–23]. Later, Akhtari first developed a new functional MRI (fMRI) method by combining AMT and MRI for localization research on epileptic foci [24].

We engineered AMT and anti-IL-1β mAb chelated to SPIONs, which were designed to have a diameter of 20 nm. To reach the epileptogenic stage and perform targeted therapy against epilepsy, we took advantage of the active targeting function of AMT for epilepsy foci to deliver anti-IL-1β mAb across the BBB.

## Methods

### Superparamagnetic iron oxide magnetic nanoparticles (SPIONs)

We followed a process similar to that of Akhtari [24] and Fu [25] to generate three types of superparamagnetic iron oxide magnetic nanoparticles (plain-SPIONs, anti-IL-1β-mAb-SPIONs and AMT-anti-IL-1β-mAb-SPIONs). The nanoparticles were surrounded by a polyethylene glycol (PEG) layer coating. The core of the nanoparticles was formed from monocrystalline iron oxide cores of maghemite. The average diameter of the nanoparticles was 10–20 nm, and the average diameter of the iron oxide core was 2–3 nm. AMT and anti-IL-1β mAb were then chelated to plain particles with the same properties (solid content: 5 mg/ml; iron concentration: 2.4 mg/ml; antibody concentration: 10 μg/mg Fe; and AMT density: 5 nmol/mg Fe). Because the process of producing nanoparticles is complicated, we bought the three nanoparticle types that were used for this study from the Micromod Company (Rostock, Germany).

### Experimental animals

Forty-five male Sprague-Dawley (SD) rats (8 weeks, weighing 200–250 g) were purchased from the LuKang Pharmaceutical Co. (Shandong, China). All rats were fed under strict sterile laminar flow in the animal laboratory in separate rooms, and maintained under a 12 h light/dark cycle, with a room temperature of 25 °C and air humidity of 50%, and free access to food and water. The experiments were based on the National Institutes of Health Guide for the Care and Use of Laboratory Animals (NIH Publications No. 8023, revised 1978). The Jining Medical University Animal Ethics Commission (Shandong, China) specifically approved this study and supervised all experiments. After the laboratory testing was completed, the rats were euthanized with ketamine at 10 mg/kg via intramuscular injection (Sigma-Aldrich, St. Louis, MO, USA).

### Constructing model animals

The rats were first administered with lithium chloride (127 mg/kg, Sigma-Aldrich) by intraperitoneal (i.p.) injection, followed by atropine (1 mg/kg, Sigma-Aldrich; i.p.) 18–20 h later and then pilocarpine (270 mg/kg, Sigma-Aldrich, ip). The rats were observed for 30 min and scored according to a modified Racine seizure scale (Racine, 1972). If the rats did not reach Racine grade IV–V seizures, pilocarpine injections were repeated once every 10 min was administrated until status epilepticus onset, with a maximum additional dose of 60 mg/kg. Seizure levels with Racine grades IV–V and seizures lasting more than 1 h were considered to indicate successful TLE model development. Diazepam was given after 1 h to end the epilepsy episodes after 1 h.

### Experimental grouping

Thirty successful and surviving epileptic model rats were randomly assigned to the plain-SPIONs group, the anti-IL-1β-mAb-SPIONs group and the AMT-anti-IL-1β-mAb-SPIONs group.

### MRI studies

The rats were anaesthetized using an intraperitoneal injection of chloral hydrate (0.3–0.4 ml/100 mg, Sigma-Aldrich). After the anaesthesia, the rats were positioned and fixed above the 3-inch coil so that the centre of the coil matched the centre of the rat body and the magnetic field. The MRI scans (3.0T Siemens Magnetom, version 3.0 T; Berlin, Germany, 2500TR/70TE, repetition time (TR): 2500 ms, echo time (TE): 70 ms, T2 sequences and T2 map sequences, 6 echoes, 192*192; slices, 12; thickness slice, 2.0 mm; field of view (FOV), 80 mm; and acquisition time, 8.25 min) were acquired at two time points. We obtained the MRI images at 48 h after successful establishment of the epilepsy model and then injected the three nanoparticles (plain-SPIONs, anti-IL-1β-mAb SPIONs, and AMT-anti-IL-1β-mAb-SPIONs, 15 mg/kg) through the tail vein and acquired the MRI images at 4 h after nanoparticle injection to compare the differences between the images. A T2 map was scanned in the position of the T2 sequence, and then we used the Siemens dedicated post-processing station to measure the T2 value according to the T2 map image. The regions of interest selected for the T2 values were the bilateral temporal lobe high signal lesion areas.

### Tissue processing

We selected 5 rats randomly from each group. The rats were anaesthetized and fixed with 0.9% saline and 4% paraformaldehyde after the MRI study. The brain tissues were placed in 4% paraformaldehyde at 4 °C overnight to be fixed, followed by incubation in 30% sucrose solution at 4 °C until the tissues reached the bottom. The brains containing the entire temporal lobe were cut on a freezing sliding microtome into consecutive coronal slices at a thickness of 5 μm. The sections were stored at − 80 °C until they were ready for tissue staining. A total of 8 slices from each specimen at intervals of 6 slices were taken and randomly selected two temporal lobe slices were randomly selected for Perl’s iron staining, Nissl staining, and two types of immuno-fluorescence staining. The hippocampi were extracted from the remaining rats, and the remaining hippocampus material was used for western blotting.

### Perl’s iron stain

These steps were performed in accordance with the Perl’s iron staining kit (Solarbio Beijing, China). The frozen slices were rewarmed at 4 °C for 30 min and dried. Then, the slices were rinsed for 2 min with distilled water, dipped for 20 min into Perl’s stain, and counterstained with Nuclear Fast Red for 10 min. The slices were then rinsed for 3 s in tap water and subjected to a conventional ethanol dehydration followed by xylene transparency and a neutral balata fixation. Image acquisition was performed with an ordinary microscope (Carl Zeiss A1, Jena, Germany).

### Immunofluorescence

Frozen sections were rewarmed at 4 °C for 30 min, and 5% goat serum was used to block the slices for 2 h. Then, the sections were incubated with Cy3-labeled GFAP (1:400 dilution; Abcam, Shanghai, China) and Iba1 (1:400 dilution; Wako, Japan)/NF-κB p65 (1:500 dilution; Abcam, Shanghai, China), followed by washing three times with PBS for 10 min each time. Alexa Fluor 488 donkey anti-rabbit IgG antibody (1:1000 dilution; Abcam, Shanghai, China) was added and then the slices were incubated for 2 h in the dark, followed by washing in PBS 3 times for 10 min each, incubation in DAPI for 10 min at room temperature in the dark, and then washing in PBS 3 times at 10 min per time. Fluorescence decay was used to seal the tablets, and the images were collected using a laser scanning confocal microscope (Carl Zeiss I800, Jena, Germany).

### Nissl staining

The frozen slices were rewarmed at 4 °C for 30 min and dried before being dipped into 0.5% toluidine blue solution for 10 min (Solarbio, Beijing, China) at 60 °C. The slices were then rinsed in distilled water and subjected to conventional ethanol dehydration and xylene transparency, followed by neutral balata fixation. Image acquisition was performed using an ordinary microscope (Carl Zeiss A1, Jena, Germany).

### Western blotting

Protein samples were obtained from hippocampus tissue that was removed over dry ice in PMSF and protein lysis buffer (Beyotime Institute of Biotechnology, Jiangsu, China). The total proteins (30 µg per lane) were separated by SDS-PAGE and transferred to polyvinylidene fluoride (PVDF) membranes via electroblotting. The membrane was blocked in 5% non-fat milk containing Tween-TBS (TBST) for 1 h at room temperature, followed by incubation with IL-1β (1:500 dilution; Santa Cruz Biotechnology, USA) and NF-κB p65 (1:500 dilution; Abcam, Shanghai, China) primary antibodies at 4 °C overnight for immunoblotting.

After rinsing in TBST, the membranes were incubated with the secondary antibody, the horseradish peroxidase (HRP)-conjugated goat anti-rabbit IgG (1:5000 dilution; Santa Cruz Biotechnology, USA) secondary antibody for 1 h at room temperature. Protein bands were visualized using ECL western blot detection reagents (ECL, Beyotime Institute of Biotechnology) to detect HRP activity and Image Quant LAS 500 (General Electric Company, USA) was used to capture the band image densities. Optical density was determined and then normalized them to the corresponding amounts of β-actin.

### Statistical analysis

All the analyses were performed with SPSS 21.0 software. All the data are expressed as mean ± SD. Multiple comparisons among groups were performed by one-way analysis of variance, and comparisons between two groups were performed using the LSD test. T-tests were used for the pre/post injection comparison of nanoparticles. A p < 0.05 was considered statistically significant.

## Results

### TLE-model induction

The SD rats began to display the expected defecation and urination, somatic shaking, and stereotyping at 10 min after intraperitoneal pilocarpine injection until eventually experiencing generalized tonic–clonic seizures. The success percentage of the model was 75.6% (of 45 total rats, 40 displayed successful acute TLE; 6 died and 34 survived; 30 rats were used for this study, and the remaining 4 rats were used for practising the experimental procedures such as tissue processing).

### MRI studies

**Fig. 1:**
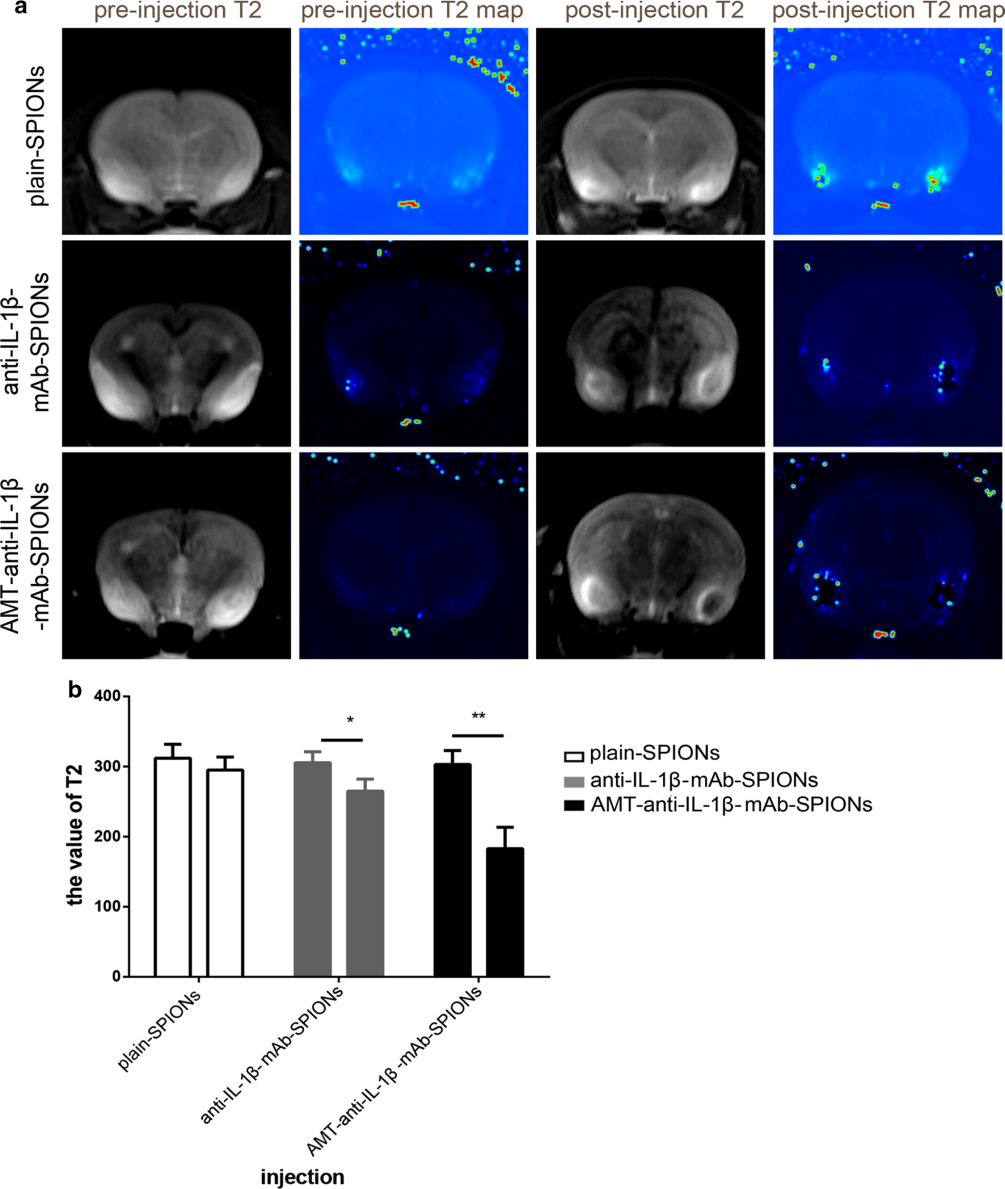
The images of MRI studies and the corresponding statistical drawing. T2-weighted MRI images show high signal lesions in the brains of the acute TLE models. The T2-weighted MRI signal intensity was slightly reduced after plain-SPION injection. The T2-weighted MRI signal intensity was decreased after anti-IL-1β-mAb-SPION injection. The T2-weighted MRI signal was significantly decreased after AMT-anti-IL-1β-mAb-SPION injection (**a**). The statistical analysis result was consistent with the images from MRI studies (**b**, *p < 0.05, **p < 0.01). There was no significant difference in the values of the T2 signals compared with those obtained pre-injection (**b**, p > 0.05). There was a significant difference in the T2 signal value after injection of the three types of particles (**b**, *p < 0.05, **p < 0.01)

To study the images of the three types of magnetic nanoparticles, we performed MRI studies. The resulting T2-weighted MRI images showed high signal lesions in the brains of the acute TLE models (Fig. 1a). There was no significant difference in the values of the T2 signal between the three groups before particle injection (p > 0.05, Fig. 1b). The T2-weighted MRI signal intensity was slightly reduced after plain-SPIONs injection (Fig. 1a), and there was no significant difference in the values of the T2 signals in comparison with those obtained pre-injection (p > 0.05, Fig. 1b). The T2-weighted MRI signal intensity was decreased after anti-IL-1β-mAb-SPION injection (Fig. 1a), and there was a significant difference in the T2 signal values in comparison with those obtained pre-injection (p < 0.05, Fig. 1b). The T2-weighted MRI signal was significantly decreased after AMT-anti-IL-1β-mAb-SPION injection (Fig. 1a), and there was a highly significant difference in the value of the T2 signal in comparison with the pre-injection values (p < 0.01, Fig. 1b). There was also a significant difference in the T2 signal value after the three types of particle injection (*p < 0.05, **p < 0.01, Fig. 1b).

### Iron particle distribution

**Fig. 2:**
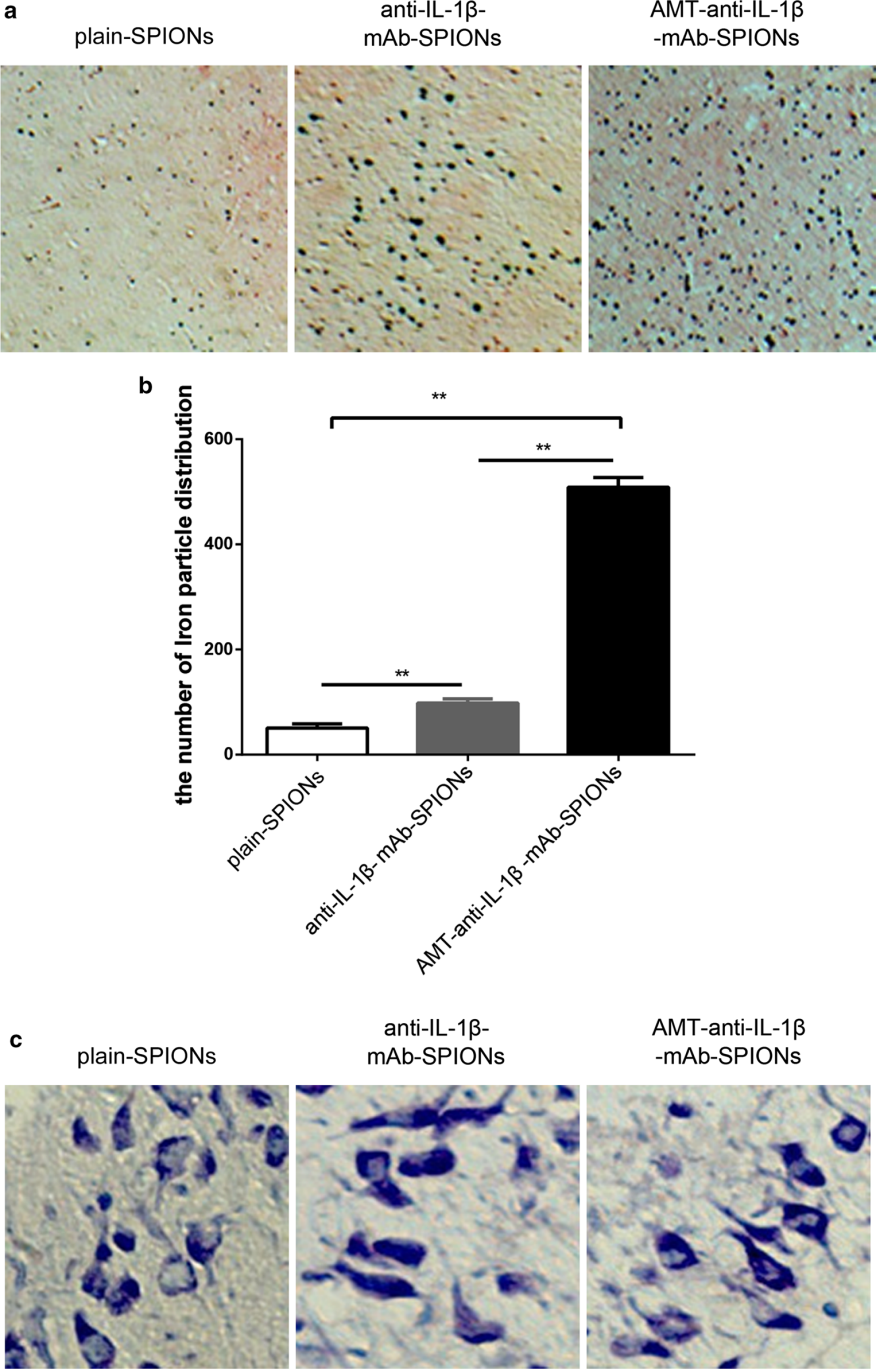
Perl’s iron staining to evaluate the iron particles and the corresponding statistical drawing. The iron particles were most densely distributed in the AMT-anti-IL-1β-mAb-SPION group (**a**), and the statistical analysis was consistent with this finding. **b** *p < 0.05, **p < 0.01, magnification ×40. Nissl staining to evaluate neuronal morphology and loss. These phenomena of Nissl bodies shrank, and the abnormal neuronal morphology was improved but not very obvious after injection of AMT-anti-IL-1β-mAb-SPIONs than after injections of anti-IL-1β-mAb-SPIONs and plain-SPIONs (**c**). Magnification ×400

We applied Perl’s iron staining to study the distribution of iron particles in the rat brains. The comparison of the number of iron particles for the three types of magnetic nanoparticles showed different values between each group (Fig. 2a). The iron particles were most densely distributed in the AMT-anti-IL-1β-mAb-SPION group (Fig. 2a). The iron particle distribution of the three types of magnetic nanoparticles also showed significant differences among the groups (**p < 0.01, Fig. 2b). The iron particle distribution of the three magnetic nanoparticle types was consistent with the T2-weighted MRI signal intensity findings.

### Pathological section staining analysis

**Fig. 3:**
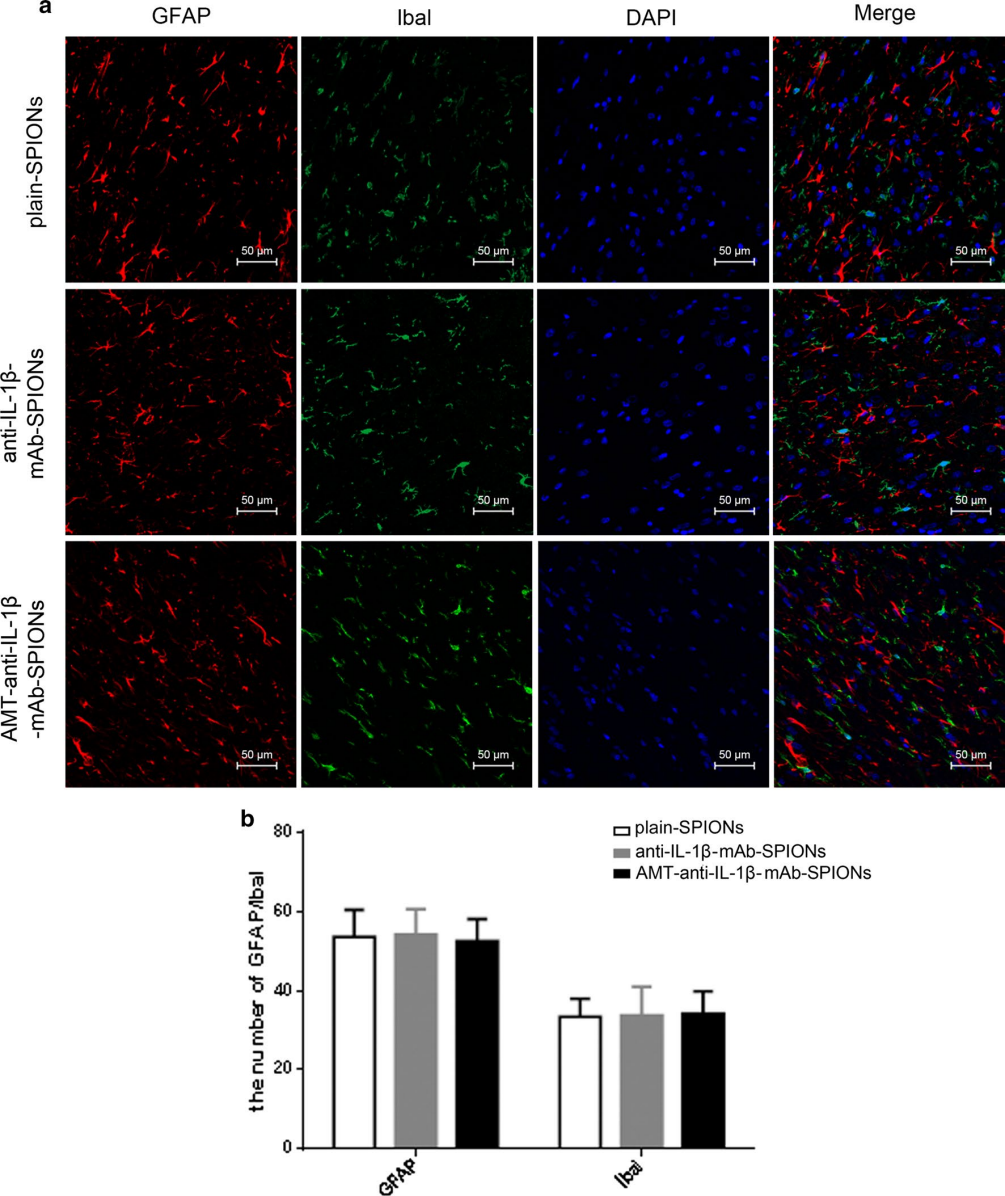
Immunofluorescence staining of GFAP and Ibal and the corresponding statistical drawing. Astrogliosis and microglial activation involving cell hypertrophy and cell proliferation were assessed in three groups (**a**), and there was no significant difference among the groups (p > 0.05, **b**). Magnification ×200

Nissl staining and immunofluorescence staining were applied to study the influence of the three magnetic nanoparticle types on neurons, astrocytes and microglia cells. The Nissl bodies shrank, and the neurons showed abnormal morphology, with small cell sizes and triangular or irregular shapes; in addition, the boundaries of the nucleus and cytoplasm were unclear (Fig. 2c). These alterations were improved after injecting AMT-anti-IL-1β-mAb-SPIONs, but this effect was not obvious in comparison with the injections with anti-IL-1β-mAb-SPIONs and plain-SPIONs (Fig. 2c). Astrogliosis (Fig. 3a) and microglial activation (Fig. 3a) involving cell hypertrophy and cell proliferation were assessed by laser scanning confocal microscopy, there was no significant difference among the three groups (p > 0.05, Fig. 3b).

### Inhibitory effect on inflammation by the IL-1β monoclonal antibody

**Fig. 4:**
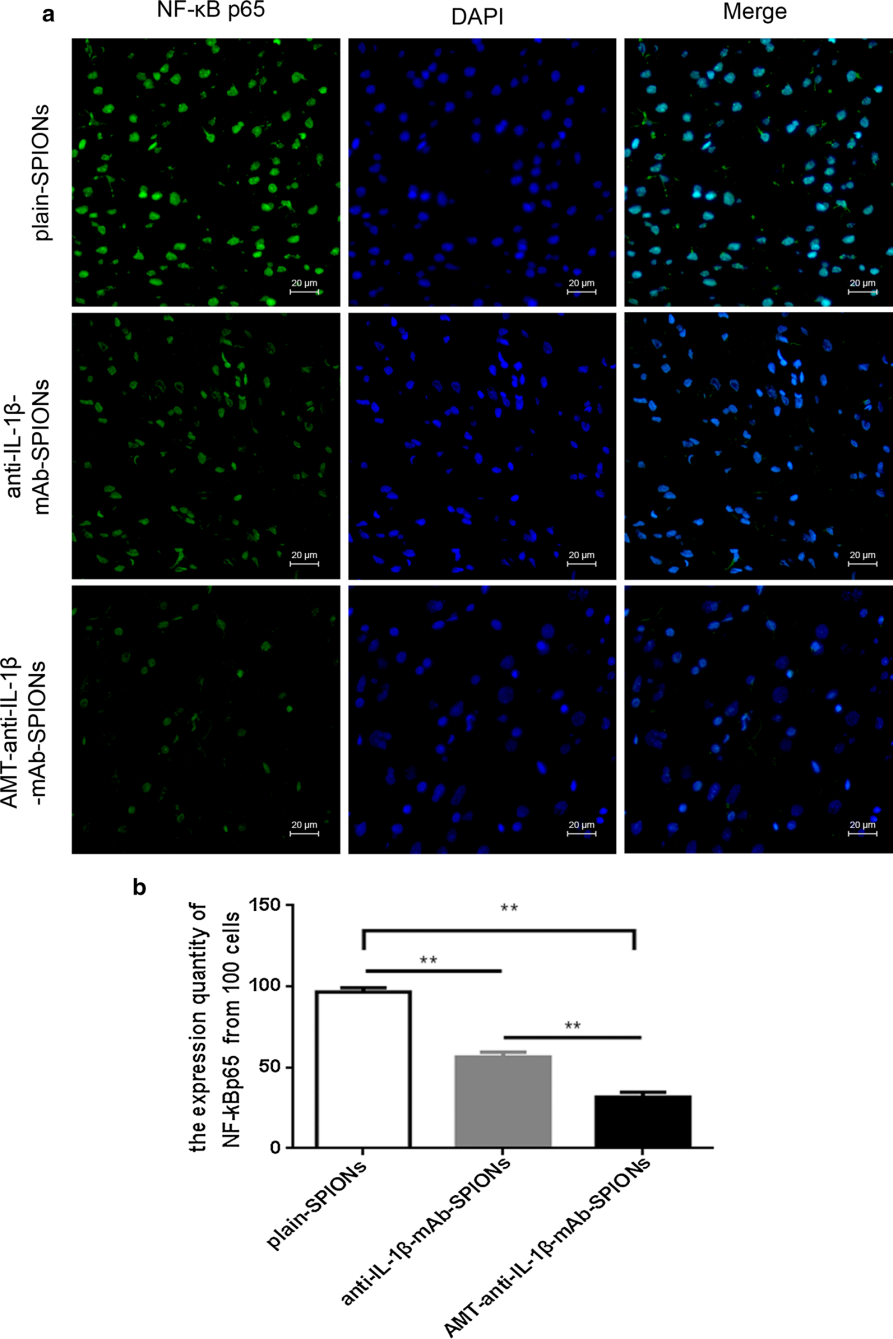
Immunofluorescence staining of NF-κBp65 expression. NF-κBp65 was primarily expressed in the nucleus but barely expressed in the cytoplasm of the cells. The expression of NF-ĸBp65 was more clearly decreased in the AMT-anti-IL-1β-mAb-SPION group than that in the anti-IL-1β-mAb-SPION and plain-SPION groups (**a**). The corresponding statistical analysis showed significant differences in the NF-ĸB expression rate among the groups (**p < 0.01, **b**). Magnification ×400

To detect the inhibitory effects of particle treatment on IL-1β and NF-κB, immunofluorescence and western blotting were used in this study. We studied the expression of NF-ĸBp65 in the brain of TLE rats after injecting the three particle types. NF-κBp65 was primarily expressed in the nucleus, but barely expressed in the cytoplasm, of the cells (Fig. 4a). The expression of NF-ĸBp65 was more clearly decreased in the AMT-anti-IL-1β-mAb-SPION group in comparison with the anti-IL-1β-mAb-SPION and plain-SPION groups based on the immunofluorescence method (Fig. 4a). Corresponding statistical analysis showed significant differences of NF-ĸB expression rate among the groups (**p < 0.01, Fig. 4b).

**Fig. 5:**
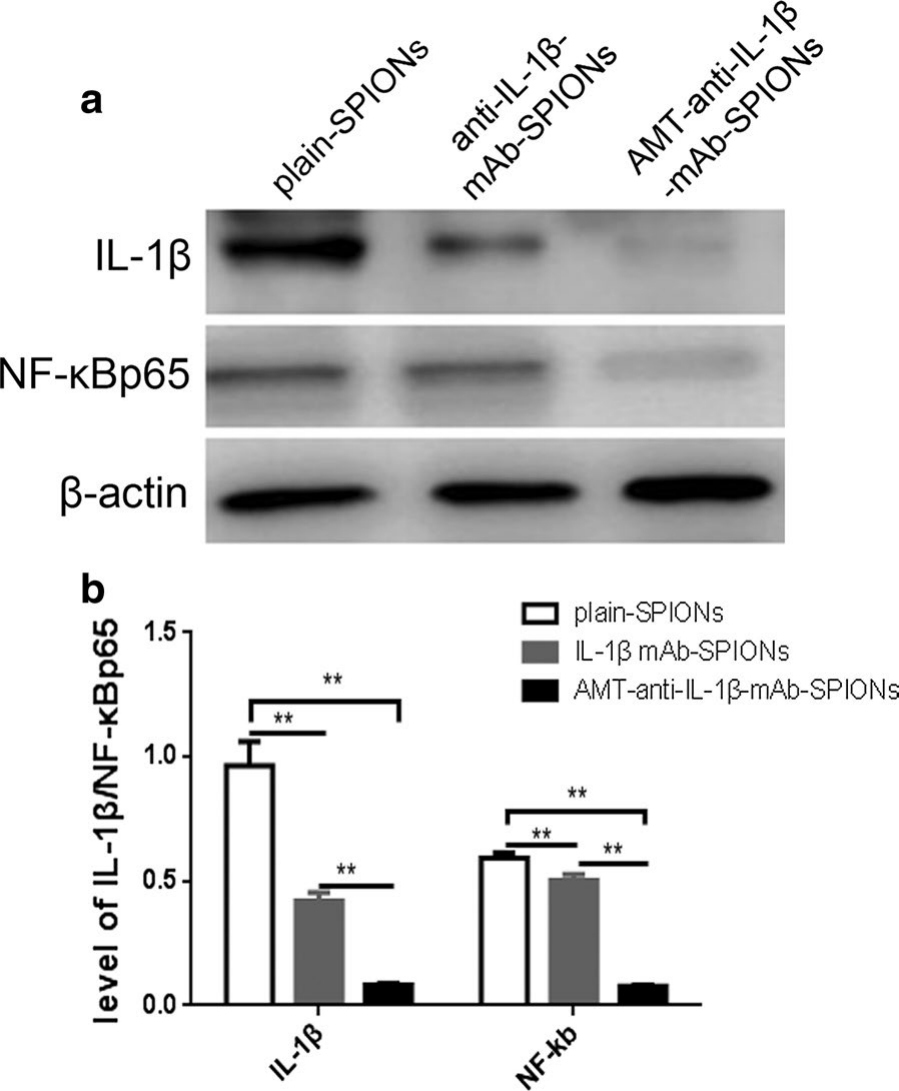
Western blot exposure imaging and the corresponding statistical drawing. We normalized IL-1β and NF-ĸB p65 to the corresponding amounts of β-actin. The IL-1β and NF-ĸB p65 concentrations were more clearly decreased in the AMT-anti-IL-1β-mAb-SPION group than those in the anti-IL-1β-mAb-SPION and plain-SPION groups (**p < 0.01, **a**, **b**)

Western blot analysis detected IL-1β and NF-κB p65 expression in the rat hippocampus tissues. The IL-1β and NF-ĸB p65 concentrations were reduced in the rats that were treated with anti-IL-1β-mAb-SPIONs and AMT-anti-IL-1β-mAb-SPIONs in comparison with the rats that were treated with plain-SPIONs (Fig. 5a). Furthermore, the inhibition observed in the AMT-anti-IL-1β-mAb-SPION group was more obvious, and a significant difference was observed among the three groups (p < 0.01, Fig. 5b).

Taken together, the immunofluorescence and western blotting results indicate that IL-1β and NF-κB p65 expression levels were decreased significantly following AMT-anti-IL-1β-mAb-SPION injections; furthermore, the results of these three approaches were consistent.

## Discussion

This study shows that the IL-1β monoclonal antibody combined with AMT and SPIONs can reduce the concentration of IL-1β more effectively due to improved location targeting The pathological mechanism of epilepsy is very complicated. It is presently believed that ion channel dysfunction, excitatory/inhibitory neurotransmitter expression imbalance, and glial cell microenvironment destruction are involved in the pathology of epilepsy. It is also well known that inflammation is important in the pathogenesis of epilepsy. In a study on resected hippocampi from TLE patients, one of the key molecular signatures of epilepsy was IL-1 [26]. Accordingly, in animal models, targeting IL-1 can reportedly reduce seizures [27, 28]. IL-1β is one of the most important inflammatory types of IL-1. Epileptic patients and rat models have demonstrated high levels of IL-1β expression in the hippocampus zone [29, 30], which was crucial for the network characteristics and neuronal excitability, making IL-1β a potential therapeutic target [31–33].

IL-1β is involved in the pathogenesis of epilepsy in a variety of ways. IL-1β promotes N-methyl-D-aspartate (NMDA)-mediated glutamate release from synaptic terminals and blocks glutamate reuptake from the synaptic space, which is mediated by astrocytes. Moreover, the activation of glutamate receptors causes an influx of a large number of sodium ions, which increases neuronal excitability [34]. IL-1β reduces GABA-mediated neurotransmission and inhibits Cl^−^ outflow, which is mediated by GABA receptors, hence reducing the suppression of signal transduction mediated by GABA receptors that contributes to the occurrence of epilepsy in TLE patients [35]. The activation of IL-1R1 induces NR2B subunit phosphorylation of the NMDA receptor complex, which is mediated by the Src kinase within minutes, leading to increased Ca^2+^ influx into neurons [36, 37]. Moreover, other studies have shown that IL-1β regulates the PI3K/Akt/mTOR signalling pathway to generate mesio-temporal lobe epilepsy (MTLE) [38]. The kynurenine pathway (KP) is an important pathway of tryptophan (TRY) metabolism in the brain. The KP is based on inflammatory factors mediated by TRY via tryptophan 2,3-dioxygenase (TDO) or indolamine-2,3-dioxygenase (IDO), which is broken down to produce kynurenine (KYN). KYN can be further broken down into quinolinic acid (QA), which is a potentially neurotoxic metabolite, and kynurenic acid (KYNA), which is a potentially neuroprotective metabolite. KYN and QA are rich in glial cells in the hippocampus of the temporal lobe, affecting neuronal activity and partial neurotransmitter expression in this area, which may be a potential factor in the onset of epilepsy. IDO is induced by proinflammatory cytokines. It is suggested that chronic epilepsy can induce the expression of inflammatory cytokines, which can, in turn, induce the expression of IDO1 in the brain. In TRY metabolism, the first step involves IDO, especially in response to inflammation [39].

Studies have suggested that IL-1β reduces hippocampal neurogenesis and activates the neurotoxic branches of the KP in humans. In addition, IL-1β promotes the proliferation of undifferentiated progenitor cells, which is associated with increased activation of KP neuroprotective branches [40].

The upregulation of IDO1 subsequently increases the KYN/TRY ratio and decreases the serotonin/tryptophan ratio in the hippocampus. Studies have observed that the induction of IL-1β and IL-6 expression induces upregulation of IDO1 expression in the hippocampus in chronic TLE rats, which was almost certainly a result of cerebral IDO induction by IL-1β [41]. The neurotoxic effects and mechanisms of QA that are the products of Kynurenine pathway which is produced by provoking enhanced intracellular calcium through over activation of NMDAR, augmenting levels of extracellular glutamate, increasing reactive oxygen species and reactive nitrogen species formation, reducing activity and expression of antioxidant systems as well as oxidative stress, stimulating protease activity, and cell death [42–45]. QA may be the primary mediator of convulsions acting through glutamate and GABA. One of the reported mechanisms of QA neurotoxicity is to increase accumulation of glutamate at the synapse by increasing its release from neurons and inhibiting its uptake by astrocytes [46].

In this study the IL-1β monoclonal antibody that was chelated to magnetic nanoparticles was beneficial for targeted epilepsy treatment. The results of this study showed that the expression of inflammatory cytokines was significantly different among the AMT-anti-IL-1β-mAb-SPION, anti-IL-1β-mAb-SPION and plain-SPION groups. Moreover, the inhibition of inflammatory cytokines was most obvious in the AMT-anti-IL-1β-mAb-SPION group. Therefore, we can conclude that the IL-1β monoclonal antibody reached an effective therapeutic concentration at the epileptogenic focus in the acute TLE model due to the active targeting of AMT and the unique advantage of magnetic nanoparticles in the acute TLE model.

In the present study, we studied the MRI images and histopathology of SD rats injected with either plain-SPIONs, anti-IL-1β-mAb-SPIONs or AMT-anti-IL-1β-mAb-SPIONs. The results showed that the magnetic field-guided delivery of plain-SPIONs, anti-IL-1β-mAb-SPIONs and AMT-anti-IL-1β-mAb-SPIONs allowed them to penetrate the BBB of rats in the acute TLE model. The reasons why these particles can enter the brain are very complicated. Seizures induce high levels of inflammatory responses, which are involved in the generation and spread of epileptic activity [47]. Previous evidence has shown that BBB permeability increases after status epilepticus [48]. On the one hand, the inflammatory reaction caused by epilepsy itself changes the function of the BBB by destroying the integrity of tight junctions and enhancing vascular endothelial cell endocytosis, which enables monocytes and macrophages in the blood to cross the BBB [49]. On the other hand, the superparamagnetic properties of SPIONs can induce a local magnetic field under the influence of an external magnetic field, strongly affecting the relaxation process of hydrogen protons in water molecules. This action effectively shortens the time of T2, leading the lesions to show negative signal enhancement. Furthermore, the small size effect of the SPIONs was beneficial in terms of leaking through the BBB [50, 51] and we could observe a change in the MRI signal after injecting the three types of nanoparticles. Previous studies have shown that nanoparticles in the tissue change the T1 and T2 signals in a concentration-dependent manner by changing the magnetic environment around the proton [52]. For instance, the T2 relaxation time of SPIONs was reduced from 170 to 130 ms in a phantom study about magnetic nanoparticles [15].

Imaging and histopathology in combination with molecular biology revealed that magnetic field-guided delivery of anti-IL-1β-mAb-SPIONs enabled the particles to enter the brain tissue, and these particles displayed much higher MRI T2 sensitivity than the plain-SPIONs. Previous evidence showed that iron oxide nanoparticles that were mediated by an antibody could penetrate the BBB through receptor-mediated transport [53]. Moreover, our laboratory previously showed that anti-IL-1β-mAb-SPIONs that were guided by a magnetic field were more likely to be taken up by brain tissue in comparison with plain-SPIONs, and these particles also significantly enhanced the anti-inflammatory effect of treatment [25]. Thus, the current study is consistent with the results of our previous study.

Imaging and histopathology in combination with molecular biology demonstrated that magnetic field-guided delivery of AMT-anti-IL-1β-mAb-SPIONs enabled the particles to enter brain tissues, and this type of particle displayed much higher MRI T2 intensity than anti-IL-1β-mAb-SPIONs or plain-SPIONs, which provided a more precise location of the epileptic focus. SPIONs were more easily absorbed by the brain parenchyma due the active targeting of AMT. As an epilepsy tracer, AMT revealed specific brain functional areas under MRI, specifically a negative enhancement signal. Studies implicated the kynurenine pathway of tryptophan metabolism as a primary mechanism of increased brain tissue retention of AMT in epileptogenic brain regions, rather than alterations in serotonin synthesis [54]. AMT was originally designed as a tracer to measure the serotonin synthesis rate. In epilepsy pathology, the kynurenine metabolic pathway is significantly enhanced, and AMT is localized to regions in the brain that synthesize serotonin. This observation implies that the kynurenine pathway of tryptophan metabolism increases the brain tissue retention of AMT in epileptogenic brain regions, instead of inducing serotonin synthesis alterations [55]. The activation of ammonia-2,3-indole dioxygenase results in a tenfold increase in brain quinolinic acid (a metabolite of the kynurenine pathway) [20, 56]. Moreover, studies have shown that the magnetic induction of the kynurenine pathway may be associated with immune activation in the pathological state of the epileptic focus [54].

Neurons and glia activate the corresponding cognate receptor to induce IL-1β release, trigger NF-ĸB inflammatory gene cascades and exert direct neuromodulatory functions in injured tissue. In this study, the results of the western blot analysis in our study showed that NF-ĸB expression was significantly decreased after the injection of AMT-anti-IL-1β-mAb-SPIONs in this study. Neurons, astrocytes and microglia contribute to inflammatory mediator synthesis [57–59]. When the BBB permeability is damaged, systemic invading leukocytes contribute to seizures [60, 61]. Neuron excitability and plasticity can be modulated by neurotransmitters, which are released by glial cells and astrocytes [62]. Astrocytes can also promote the onset of epilepsy synchronous activity in the neuronal network [63]

and this action may induce ictal discharge generation [64]. Increasing studies have found that astrocytes play a role as active partners in neural information processing. Novel techniques such as Ca^2+^ imaging and advanced electrophysiological testing have revealed that, like neurons, astrocytes have functional transmitter receptors and ion channels [65]. In cultured astrocytes, an increased level of the intracellular Ca^2+^ concentration promoted glial cells to release glutamate [66]. The activation and proliferation of microglia can also promote hyperexcitability via neuroinflammatory mechanisms and neurotoxicity [67–69].

We studied astrocytes and microglial cells with immunofluorescence; astrogliosis and microglial activation occurred in each group, mainly resulting in significant increases in GFAP and Iba1 expression levels, cell hypertrophy and cell proliferation. These phenomena were not significantly different following injection of the three particles; this result may be due to the short experimental cycle. Alternatively, perhaps there was no relevant response to change. The effects of SPIONs, anti-IL-1β-mAb-SPIONs, and AMT-anti-IL-1β-mAb-SPIONs on neurons, astrocytes and microglia were also studied in the current investigation. The neuron morphology was not clearly improved after injecting AMT-anti-IL-1β-mAb-SPIONs in comparison with the injection of anti-IL-1β-mAb-SPIONs and plain-SPIONs, which further demonstrated that IL-1β monoclonal antibodies reached an effective treatment concentration in the acute TLE model via the active targeting ability of AMT. However, the neuron morphology phenomenon was not improved significantly with treatment. Microglial activation and astrogliosis were observed but were not effectively improved after injecting AMT-anti-IL-1β-mAb-SPIONs or anti-IL-1β-mAb-SPIONs. In future work, we aim to address the chronic phase of epilepsy and related behavioural observations due to the impact of these particles on neurons, astrocytes and microglia. Furthermore, we have not yet analysed the optimal concentration of iron particles and the optimal therapeutic concentration of IL-1β monoclonal antibodies; we also did not study the toxicology of these particles. These important questions are currently under investigation by our research group.

## Conclusions

Our study shows that AMT can actively target nanoparticles to the epileptogenic focus and can accurately locate epileptic lesions through MRI due to the unique advantages of magnetic nanoparticles. The IL-1β monoclonal antibody successfully penetrated the BBB to reach effective therapeutic concentrations in epileptic foci due to the precise targeting location of AMT and MRI.

## Abbreviations

TLE: temporal lobe epilepsy
AMT: alpha-methyl-l-tryptophan
MTDS: magnetic targeting drug delivery system
SPIONs: superparamagnetic iron oxide nanoparticles
anti-IL-1β mAb: IL-1β monoclonal antibody
BBB: blood brain barrier
MRI: magnetic resonance imaging
IL-1β: Interleukin-1β
TSC: tuberous sclerosis complex
PET: positron emission tomography
fMRI: functional MRI
PEG: polyethylene glycol
SD: Sprague–Dawley
KP: kynurenine pathway
TRY: tryptophan
TDO: tryptophan 2,3-dioxygenase
IDO: indolamine-2,3-dioxygenase
KYN: kynurenine
QA: quinolinic acid
KYNA: kynurenic acid
